# Pyridostigmine Improves the Effects of Resistance Exercise Training after Myocardial Infarction in Rats

**DOI:** 10.3389/fphys.2018.00053

**Published:** 2018-02-12

**Authors:** Daniele J. Feriani, Hélio J. Coelho-Júnior, Juliana C. M. F. de Oliveira, Maria A. Delbin, Cristiano T. Mostarda, Paulo M. M. Dourado, Érico C. Caperuto, Maria C. C. Irigoyen, Bruno Rodrigues

**Affiliations:** ^1^Human Movement Laboratory, Universidade São Judas Tadeu, São Paulo, Brazil; ^2^Faculty of Physical Education, Universidade Estadual de Campinas, Campinas, Brazil; ^3^Department of Structural and Functional Biology, Institute of Biology, Universidade Estadual de Campinas, Campinas, Brazil; ^4^Faculty of Physical Education, Universidade Federal do Maranhão, São Luís, Brazil; ^5^Heart Institute (InCor), Faculdade de Medicina da Universidade de São Paulo, São Paulo, Brazil

**Keywords:** myocardial infarction, resistance training, pyridostigmine bromide, autonomic modulation, inflammation

## Abstract

Myocardial infarction (MI) remains the leading cause of morbidity and mortality worldwide. Exercise training and pharmacological treatments are important strategies to minimize the deleterious effects of MI. However, little is known about the effects of resistance training combined with pyridostigmine bromide (PYR) treatment on cardiac and autonomic function, as well as on the inflammatory profile after MI. Thus, in the present study, male Wistar rats were randomly assigned into: control (Cont); sedentary infarcted (Inf); PYR – treated sedentary infarcted rats (Inf+P); infarcted rats undergoing resistance exercise training (Inf+RT); and infarcted rats undergoing PYR treatment plus resistance training (Inf+RT+P). After 12 weeks of resistance training (15–20 climbs per session, with a 1-min rest between each climb, at a low to moderate intensity, 5 days a week) and/or PYR treatment (0.14 mg/mL of drink water), hemodynamic function, autonomic modulation, and cytokine expressions were evaluated. We observed that 3 months of PYR treatment, either alone or in combination with exercise, can improve the deleterious effects of MI on left ventricle dimensions and function, baroreflex sensitivity, and autonomic parameters, as well as systemic and tissue inflammatory profile. Furthermore, additional benefits in a maximal load test and anti-inflammatory state of skeletal muscle were found when resistance training was combined with PYR treatment. Thus, our findings suggest that the combination of resistance training and PYR may be a good therapeutic strategy since they promote additional benefits on skeletal muscle anti-inflammatory profile after MI.

## Introduction

Myocardial infarction (MI) is defined as the myocardial cell death due to prolonged ischemia. MI is the most prevalent kind of cardiovascular disease (CVD) and one of the leading causes of death worldwide; undoubtedly, making this condition a serious public health concern (Mozaffarian et al., 2015). After MI, local changes are observed in cardiac tissue, such as ventricular remodeling characterized by left ventricular dilatation, alterations in ventricular wall structure, increase in the remaining muscle mass and a decrease in cardiac function (Grans et al., 2014). In addition, systemic changes are frequently evoked after MI, and a dysregulation of the autonomic system through the imbalance between parasympathetic and sympathetic activity modulation can be observed (Rodrigues et al., 2014).

Nevertheless, autonomic dysfunction is not the only systemic phenomenon induced by MI. Indeed, cardiac injury triggers the activation of the immune system, which in turn leads to detrimental effects on the cardiac and other tissues (e.g., skeletal muscle) (Nian et al., 2004). An altered inflammatory profile is one the most frequent characteristic triggered by MI. Such alterations are represented by a marked increase in proinflammatory cytokines, such as tumor necrosis factor-α (TNF-α) and interleukins (ILs), along with a null or a slight decrease in plasma levels of anti-inflammatory cytokines, e.g., interleukin-10 (IL-10) (Kaur et al., 2006; Kalogeropoulos et al., 2012).

Exercise training, particularly aerobic exercise, has demonstrated a great potential to counteract the deleterious effects of MI on the organic system. In fact, a number of studies have indicated that exercise training programs based on aerobic exercise may elicit improvements in several parameters commonly affected by MI, including, but not limited to, cardiac morphology and function, exercise tolerance, autonomic function, and inflammatory state (Rondon et al., 2006; Flores et al., 2010; Jorge et al., 2011). Moreover, the findings of La Rovere et al. (2002) have suggested that MI patients submitted to a short-term aerobic exercise training present increased long-term survival.

On the other hand, resistance training (RT), a modality of exercise training in which dynamic or static muscle contraction is resisted by a predetermined load, has been little explored in the context of MI. Beneficial effects on primary clinical parameters (e.g., blood pressure), cardiovascular risk factors (e.g., triglycerides), and parallel conditions involving other CVDs (e.g., dynapenia and muscle atrophy) have been largely demonstrated in the literature (Cornelissen et al., 2011; Moraes et al., 2012). Most recently, the research was undertaken by Grans et al. (2014) on the effects of RT on MI has been particularly promising. However, more evidence in animal models is still necessary to start the design of clinical studies.

When characterized by an increased sympathetic activity, autonomic dysfunction becomes one of the key elements in the pathophysiology of MI, as it contributes to poor prognosis (La Rovere et al., 1998; Toschi-Dias et al., 2017). On the other hand, researchers have investigated whether an increased concentration of acetylcholine (ACh), the neurotransmitter released from the endings of parasympathetic nerve fibers, might be mimicking an increased activity of the parasympathetic branch of the autonomic nervous system, thus reducing the deleterious effects of the autonomic dysfunction on the organic system.

In this sense, several studies have demonstrated positive evidence for treatment with pyridostigmine bromide (PYR), a medication that inhibits acetylcholinesterase activity, and may play a role in the reestablishment of cardiac autonomic function to pre-infarction levels (La Fuente et al., 2013; Lataro et al., 2013; Durand et al., 2014; Corrêa et al., 2015; Rocha et al., 2016). PYR treatment may also improve autonomic balance (La Fuente et al., 2013; Lataro et al., 2013; Durand et al., 2014; Rocha et al., 2016), and baroreflex sensitivity (La Fuente et al., 2013), as well as decrease the inflammatory state (Rocha et al., 2016; Feriani et al., 2017) of infarcted rats. Interestingly, a recent study of our group has demonstrated that the combination of PYR treatment and aerobic exercise could elicit superior effects on autonomic modulation and inflammatory profile of infarcted rats to physical exercise or PYR treatment alone (Feriani et al., 2017). However, the combination of a pharmacological treatment and exercise has not been studied using RT.

Therefore, this study aimed to investigate the effects of 3 months of an RT protocol and PYR treatment, alone, and combined, on physical capacity, cardiac morphometry, and function, hemodynamic, and autonomic parameters, as well as on systemic and tissue inflammatory profile of infarcted rats.

## Materials and methods

The experimental protocol was approved by the Institutional Animal Care and Use Committee of the São Judas Tadeu University (permit number: 008/2013) and the study was conducted in accordance with the Guide for the care and Use of Laboratory Animals, issued by the National Institutes of Health (NIH Publication number 96-23, revised in 1996).

Male Wistar rats (250–300 g) were obtained from the Animal House of the São Judas Tadeu University, São Paulo, Brazil. Rats were fed standard laboratory chow and water ad libitum and were housed in collective polycarbonate cages, in a temperature-controlled room (22°C) under a 12 h dark–light cycle (lights on 07:00–19:00 h).

Rats were randomly assigned into 5 groups: control (Con, *n* = 9); sedentary infarcted (Inf, *n* = 10); sedentary infarcted treated with PYR (Inf+P, *n* = 9); infarcted rats submitted to resistance exercise training (Inf+RT, *n* = 9); and infarcted rats undergoing PYR treatment plus resistance training (Inf+RT+P, *n* = 9). Rats allocated to the Con, Inf, and Inf+RT groups had unlimited access to drinking water, whereas those in the Inf+P and Inf+RT+P groups had similar access to water containing PYR (0.14 mg/mL; Sigma, St Louis, MO, USA), as described previously (La Fuente et al., 2013; Feriani et al., 2017). The solutions containing PYR were prepared daily, and the water pot was wrapped in a black paper. PYR treatment started immediately after MI surgery and continued for 3 months after this procedure. Water consumption was monitored during the experimental period in the PYR-treated and untreated groups.

### Experimental design

At the beginning of the protocol, 1 day after MI surgeries, the animals underwent echocardiographic evaluation and were then included in the current study if they exhibited an MI size between 30–50% of the left ventricular wall and/or ejection fraction < 30%. Subsequently, rats underwent a maximum load test (MLT) 2 days after MI surgery (i.e., Initial); 45 days following the beginning of the exercise training to adjust the training load (i.e., Middle; data not shown), and 1 day after the end of the 3-month exercise program (i.e., Final). The animals were treated with PYR and/or underwent resistance exercise training for 3 months. One day after the last MLT, a new echocardiographic evaluation was performed in all experimental groups. On a subsequent day, after being anesthetized (80 mg/kg ketamine and 12 mg/kg xylazine, i.p.), the animals had arteries and veins catheterized for hemodynamic assessment, baroreflex sensitivity (BrS), autonomic tone, and spectral analyses measurements. One day after the last evaluation, the animals were killed by decapitation in order to remove the plasma, left ventricle, and left soleus skeletal muscle to evaluate the inflammatory profile. Time course of the experimental design used in this study is detailed in the Supplementary Material 1.

### Myocardial infarction

Anesthetized rats (80 mg/kg ketamine and 12 mg/kg xylazine, i.p.) underwent surgical occlusion of the left coronary artery, which resulted in MI, as described previously (Rodrigues et al., 2014). Briefly, after intubation, animals were positive-pressure ventilated with room air at 2.5 mL, 65 strokes/min with a pressure-cycled rodent ventilator (Harvard Apparatus, Model 683, Hox'lliston, MA, USA). For induction of MI, a 2-cm left lateral thoracotomy was performed in the third intercostal space, and the left anterior descending coronary artery was occluded with a nylon (6.0) suture at ~1 mm from its origin below the tip of the left atrium. The Con animals underwent the same procedures except for myocardial ischemia, which was not induced (Sham surgery). The chest was closed with a silk suture.

### Echocardiographic evaluation

Echocardiographic measurements were performed by a blinded observer, following the guidelines of the American Society of Echocardiography. Rats were anesthetized (80 mg/kg ketamine and 12 mg/kg xylazine, i.p.), and images were obtained using a 10–14 MHz linear transducer in a SEQUOIA 512 (Acuson Corporation, Mountain View, CA, USA) from a short-axis view at the level of the papillary muscles. One day after MI or sham surgeries (Baseline assessments), and at the final (3 months of treatment and/or exercise training), echocardiographic measurements were performed for measurements of the following parameters: left ventricular mass (LV mass); left ventricular end-diameter during diastole (LVDD); relative wall thickness (RWT); fractional shortening (FS); E wave A wave ratio (EA, as described in detail elsewhere (Rodrigues et al., 2014).

Through midtransversal and apical transversal views, MI size was measured by bi-dimensional echocardiogram. In diastole, three measurements of the endocardial perimeter (EP) and the length of the infarcted segment (ISe) were obtained for each view. MI size for each segment (ISi) was calculated by the equation ISi (%) = ISe/EP × 100. The estimation of the infarct size of each animal was calculated as the mean of ISi (%) of the 3 segments. MI size was defined as increased echogenicity and/or change in myocardial thickening or systolic movement (hypokinesia, akinesia, or dyskinesia). Our group and other researchers have previously shown strong correlations between the MI area assessed by echocardiogram and post-mortem histological analysis (dos Santos et al., 2008; Jorge et al., 2011; Feriani et al., 2017).

### Maximum load test (MLT) and resistance training

All groups underwent the MLT protocol, which was performed in a ladder adapted for rats, with 54 vertical steps 0.5 cm apart from each other. The animals were gradually adapted to climbing for 4 consecutive days prior the MI surgery. The adaptation period consisted of climbing the entire length of the ladder without any load. This period was used to familiarize the animals with the ladder and the laboratory environment. The test took place 2 days after MI surgery and consisted of an initial load of 75% of body weight, which was progressively increased with an additional 15% of body weight on subsequent climbs until the animal could not climb the entire length of the ladder after 3 attempts (muscle failure), as previously described (Grans et al., 2014; Sanches et al., 2014; Neves et al., 2016). MLT was considered as the heaviest load that the animal could successfully endure before muscle failure. 45 days after the onset of exercise training (i.e., Middle; data not shown) and 1 day after the end of the 3-month exercise program (i.e., Final), MLT was performed again to adjust the load and to analyze the effectiveness of physical training, respectively. For these MLTs (i.e., Middle and Final), the initial load was based on the previous MLT recorded. Therefore, in the MLT performed to adjust the load (i.e., Middle), the rats started the test with the maximum load achieved in the Initial test; similarly, the MLT achieved in the Middle test was used as the initial load to start the Final MLT test. The progression of the test followed a progressively increase in load (15% of initial test load until muscle failure, as above-mentioned. RT protocol was performed 5 days a week, 15–20 climbs per session, with a 1-min rest between each climb, from a low to a moderate intensity (40–60% of the MLT) for 3 months (Sanches et al., 2014), as recommended for patients with CVD (Williams et al., 2007).

### Hemodynamic, baroreflex sensitivity, and autonomic tonus assessments

At the end of the protocol, 2 catheters filled with 0.6 mL heparinized saline solution (the solution was prepared with 0.9 mL of saline and 0.1 mL of heparin) and were implanted into the carotid artery and jugular vein. The catheters were exteriorized on the back of the neck, between the scapulae of the rat, while the animals were anesthetized (80 mg/kg ketamine and 12 mg/kg xylazine, i.p.) for direct measurements of arterial pressure (AP) and vasoactive drug administration, respectively. Rats were studied 1 day after catheter placement; they were conscious and allowed to move freely during the experiments. The catheters were flushed with a 0.6 mL heparinized saline solution before the experiments. The arterial cannula was connected to a strain-gauge transducer (Blood Pressure XDCR; Kent Scientific, Torrington, CT, USA), and AP signals were recorded over a 30-min period by a microcomputer equipped with an analog-to-digital converter board (WinDaq, 2 kHz, DATAQ, Springfield, OH, USA). The recorded data were analyzed on a beat-to-beat basis to quantify changes in mean AP and heart rate (HR) (Feriani et al., 2017).

A sequential bolus injection of increasing doses of phenylephrine (PHE: 0.25–32 μg/kg) and sodium nitroprusside (SNP: 0.05–1.6 μg/kg) were given to induce at least 4 pressure responses (for each drug) ranging from 5 to 40 mmHg. A 3–5 min interval between doses was necessary for AP to return to baseline. Peak increases or decreases in mean AP after PHE or SNP injection and the corresponding peak reflex changes in HR were recorded for each dose of the drug. BrS was evaluated by a mean index, calculated by the ratio between changes in HR and changes in mean AP, allowing a separate analysis of bradycardic (BR) and tachycardic responses (TR). The mean index was expressed as bpm/mmHg, according to a previous study (Feriani et al., 2017).

After BrS measurement, the catheters were flushed with saline solution and, subsequently, AP and HR were continuously recorded at basal state. Following this procedure, animals were injected (0.2 mL) with methylatropine (3 mg/kg, i.v.).

Because the HR response to the drug reaches its peak within 3–5 min, this time interval was allowed to elapse before HR measurement. Atenolol (8 mg/kg, i.v.) was injected (0.2 mL) 10 min after methylatropine, and again the response was measured after simultaneous blockade with atenolol and methylatropine. With the experiments concluded, the catheters were kept filled with a heparinized saline solution for 24 h until the next series of measurements.

On a subsequent day, the sequence of injections was inverted (first atenolol and then methylatropine). Intrinsic heart rate (IHR) was measured after simultaneous blockade with atenolol and methylatropine. Sympathetic tonus was determined as the difference between maximum HR after methylatropine injection and IHR. Vagal tonus was calculated from the difference between the lowest HR after atenolol injection and IHR (La Fuente et al., 2013; Feriani et al., 2017).

### Cardiovascular autonomic modulation

The overall variability of the systolic AP (SAP) in time domain was assessed by the variance of the time series. Fluctuations in SAP were evaluated in the frequency domain by means of autoregressive spectral estimation. The theoretical and analytical procedures for autoregressive modeling of oscillatory components have been previously described (Malliani and Pagani, 1991; Sanches et al., 2015). Briefly, the SAP series, derived from each recording, were divided into 300 beat segments with a 50% overlap. The spectra of each segment were calculated via the Levinson-Durbin recursion and the order of the model chosen according to Akaike's criterion, with the oscillatory components quantified in low frequency (LF; 0.2–0.6 Hz) and high-frequency (HF; 0.6–3.0 Hz) ranges.

One day after *in vivo* measurements (and 5 days after last training session), the animals were killed by decapitation and blood, left ventricular and soleus skeletal muscle tissues were removed.

### Acetylcholinesterase activity

Blood samples were collected in vials containing 30 μl of EDTA (0.1 M, Sigma-Aldrich). After centrifugation (3,000 g, 4°C) for 20 min, plasma was collected and kept at −20°C until the determination of cholinesterase activity. Enzymatic assays were performed using an adaptation of the colorimetric method (Ellman et al., 1961), as described by Lataro et al. (2013) and used by our group (Feriani et al., 2017). Plasma samples (10 μl) were incubated in 96-well microplates with 0.01 M 5,5′-dithio-bis-(2-nitrobenzoic acid, DTNB, Sigma-Aldrich) and the excess of the substrate (acetylthiocholine, 0.075 M, Sigma-Aldrich) in 0.2 mM phosphate buffer, pH 8.0 at 30°C, in the presence of the selective inhibitor of butyrylcholinesterase, tetraisopropylpyrophosphoramide (IsoOMPA 10–3 M, Sigma-Aldrich). Generation of the reaction product was followed in a microplate reader (BioTek FL600, BioTek Instruments, Winooski, VT) at 405 nm for 60 min at 3-min intervals. The maximum velocity (Vmax) of the reaction for each sample was determined in duplicate and expressed as arbitrary units per minute per liter of plasma.

### Plasma, left ventricle, and soleus skeletal muscle inflammatory profile

Plasma and tissue (LV and soleus skeletal muscle) levels of proinflammatory cytokines (PICs) (tumor necrosis factor alpha [TNF-α], interferon gama [IFN-γ], interleukin-1 beta [IL-1β], interleukin-6 [IL-6]), and anti-inflammatory cytokine (interleukin-10 [IL-10]) were determined by ELISA (DuoSet ELISA, R&D Systems, Minneapolis, MN, USA) in accordance with the manufacturer's instructions. All samples were run as duplicates, the mean value was reported, and the results were normalized by LV total protein extracted by the Bradford method (Rodrigues et al., 2014; Feriani et al., 2017).

### Statistical analyses

Statistical analyses were performed using the SPSS software (Version 20.0 for Windows; SPSS Inc., Chicago, USA). Data are reported as mean ± SEM. After confirming that all continuous variables were normally distributed using the Kolmogorov-Smirnov test, statistically significant differences were assessed using a two-way ANOVA, followed by the Bonferroni post-test. Statistical differences in data measurements over time were assessed using repeated-measures ANOVA. All tests were two-sided and the significance level was established at *p* < 0.05.

## Results

### Body weight, water intake, and acetylcholinesterase activity

Initial body weights were similar between the groups (mean = 281 ± 10 g). At the end of the protocol, body weights were increased in the experimental groups when compared to their initial assessment (Con = 497 ± 10; Inf = 485 ± 15; Inf+P = 487 ± 13; Inf+RT = 465 ± 18; Inf+RT+P = 460 ± 11 g). Water consumption was similar between the experimental groups (Con = 58 ± 6; Inf = 63 ± 7; Inf+P = 55 ± 9; Inf+RT = 58 ± 10; Inf+RT+P = 62 ± 9 mL/day, respectively). Similarly, there were no differences between the ingestion of PYR between Inf+P (8 ± 3 mg/kg) and Inf+RT+P (8 ± 2 mg/kg). Acetylcholinesterase e activity (AChE) was measured in order to confirm the effectiveness of the PYR treatment. PYR-treated groups (Inf+P = 457.8 ± 37.2 and Inf+RT+P = 425.9 ± 17.6 Units/L) showed a significant reduction of AChE activity when compared to untreated groups (Con = 861.5 ± 40.2; Inf = 915.2 ± 54.3, and Inf+RT = 876.9 ± 29.5 Units/L).

### Maximum load test (MLT)

**Figure 1:**
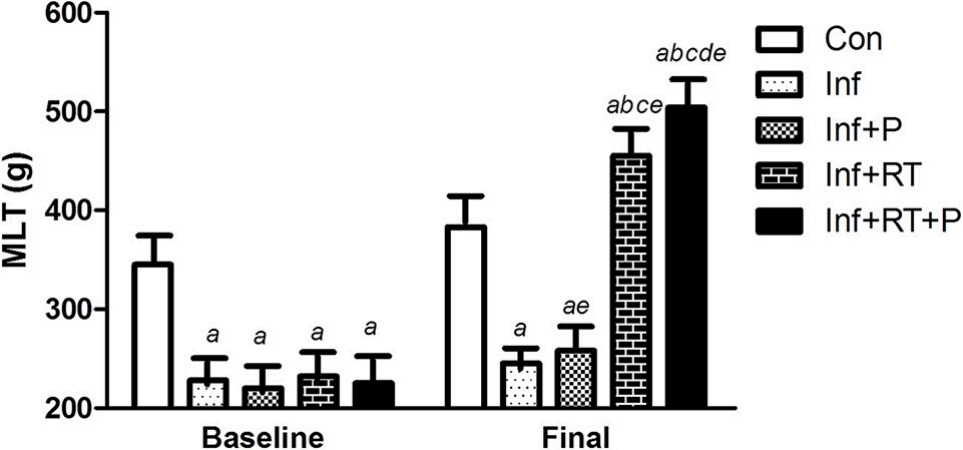
Maximal load test (MLT). Control rats (Con); Sedentary infarcted rats (Inf); Sedentary infarcted rats treated with pyridostigmine (Inf+P); infarcted rats submitted to resistance exercise training (Inf+RT); and infarcted rats submitted to treatment with pyridostigmine and resistance exercise training (Inf+RT+P); Values expressed as mean ± SEM; *a, P* < 0.05 vs. Con; *b, P* < 0.05 vs. Inf; *c, P* < 0.05 vs. Inf+P; *d, P* < 0.05 vs. Inf+RT; *e, P* < 0.05 vs. Baseline.

At the beginning of the protocol, all infarcted groups showed lower MLT when compared to Con (Figure 1). After 3 months of RT, the MLT remained lower in the Inf group when compared to Con. On the other hand, PYR treatment alone (i.e., Inf+P) elicited a slight increase in MLT when compared to baseline assessments. However, Inf+RT and Inf+RT+P groups displayed higher values when compared to Con, Inf, and Inf+P groups, as well as their baseline assessment. In addition, Inf+RT+P presented an additional increase in MLT values when compared to Inf+RT rats.

### Echocardiographic parameters

Table 1 shows the echocardiographic evaluation in experimental groups. Initially, the infarcted groups (i.e., Inf, Inf+P, Inf+RT, Inf+RT+P) showed an increased LVDD and E/A ratio, followed by a decreased EF when compared to Con group. After the experimental period, the Inf group presented an additional increase in LVDD and LV mass, and a further decrease in RTW, when compared to Con and Baseline evaluations; whereas LV mass, EF, and EA ratio remained unchanged. PYR treatment (Inf+P) elicited significant changes in the MI akinetic area, LV mass, LVDD, RWT, and EA when compared to Inf; and in EF when compared to Baseline. On the other hand, resistance training alone (Inf+RT) elicited a smaller number of alterations compared to PYR treatment (Inf+P), since only LV mass, RWT and EA ratio were increased in Inf+RT when it was compared to Inf. When the treatments were combined (Inf+RT+P), the effects of PYR treatment alone on LVDD disappeared, while significant changes in the MI akinetic area and LV mass were observed when compared to Inf+RT. No other benefits were observed when Inf+RT+P was compared to Inf+P.

**Table 1:** Echocardiographic evaluations.

		**Con**	**Inf**	**Inf+P**	**Inf+RT**	**Inf+RT+P**
MI area (%)	Baseline	–	44.4 ± 3	39.5 ± 8	40.1 ± 2	44.3 ± 4
	Final	–	45.6 ± 4	15.3 ± 2**b**	44.7 ± 5**c**	27.3 ± 2**bd**
LV mass (g)	Baseline	1.07 ± 0.02	1.15 ± 0.04	1.17 ± 0.04	1.11 ± 0.05	1.12 ± 0.06
	Final	1.00 ± 0.05	1.42 ± 0.05**ae**	1.26 ± 0.04**b**	1.55 ± 0.06**abce**	1.29 ± 0.04**abd**
LVDD (cm)	Baseline	0.62 ± 0.02	0.83 ± 0.02**a**	0.87 ± 0.05**a**	0.84 ± 0.02**a**	0.85 ± 0.06**a**
	Final	0.69 ± 0.02	0.97 ± 0.04**ae**	0.80 ± 0.01**b**	0.87 ± 0.05**a**	0.89 ± 0.04**a**
RWT	Baseline	0.39 ± 0.04	0.35 ± 0.02	0.41 ± 0.04	0.43 ± 0.05	0.44 ± 0.02
	Final	0.45 ± 0.03	0.24 ± 0.04**ae**	0.46 ± 0.04**b**	0.48 ± 0.04**b**	0.49 ± 0.02**b**
EF (%)	Baseline	40 ± 3	28 ± 2**a**	25 ± 2**a**	27 ± 3**a**	26 ± 4**a**
	Final	38 ± 1	26 ± 3**a**	34 ± 2**e**	29 ± 4	35 ± 3**e**
EA	Baseline	1.55 ± 0.11	2.77 ± 0.14**a**	2.70 ± 0.17**a**	2.75 ± 0.14**a**	2.73 ± 0.13**a**
	Final	1.57 ± 0.09	2.97 ± 0.19**a**	1.66 ± 0.15**b**	2.12 ± 0.11**b**	1.54 ± 0.17**b**

Values expressed as mean ± SEM. Two-way ANOVA with Bonferroni posttest. MI – myocardial infarction;

*LV mass, left ventricle mass; RWT, relative wall thickness; EF, ejection fraction; EA, E wave A wave ratio; a, P < 0.05 vs. Con; b, P < 0.05 vs. Inf; c, P < 0.05 vs. Inf+P; d, P < 0.05 vs. Inf+RT; e, P < 0.05 vs. Baseline*.

### Hemodynamic and baroreflex sensitivity indexes

Table 2 shows the hemodynamic function and BrS evaluation. SAP, DAP, MAP, and HR were not altered after MI or in response to any of the treatments. A significant decrease in BR was observed in all infarcted groups, reflecting the impairment of BrS. However, BR values were higher in exercise training and PYR treatment groups (i.e., Inf+P, Inf+RT, and Inf+RT+P) when compared to Inf. In turn, a decrease in TR was observed in Inf and Inf+RT when compared to Con, while TR values were higher in Inf+P and Inf+RT+P when compared to Inf, suggesting a preventive effect of PYR on this BrS index. Nevertheless, no additional differences were observed between the treated groups.

**Table 2:** Hemodynamic and Baroreflex Sensitivity parameters.

	**Con**	**Inf**	**Inf+P**	**Inf+RT**	**Inf+RT+P**
SAP (mmHg)	116 ± 8	109 ± 5	119 ± 7	124 ± 4	129 ± 9
DAP (mmHg)	78 ± 4	75 ± 8	82 ± 9	85 ± 5	87 ± 8
MAP (mmHg)	94 ± 5	88 ± 7	103 ± 9	95 ± 7	98 ± 8
HR (bpm)	327 ± 8	351 ± 12	338 ± 10	355 ± 14	319 ± 9
TR (bpm/mmHg)	3.12 ± 0.21	1.43 ± 0.24**a**	2.84 ± 0.32**b**	2.08 ± 0.17**a**	2.95 ± 0.22**b**
BR (bpm/mmHg)	−2.34 ± 0.04	−1.27 ± 0.03**a**	−2.12 ± 0.05**ab**	−2.14 ± 0.02**ab**	−2.11 ± 0.07*ab*

*Values expressed as mean ± SEM. Two-way ANOVA with Bonferroni posttest. SAP, systolic arterial pressure; DAP, diastolic arterial pressure; MAP, mean arterial pressure; HR, heart rate; TR, tachycardic response; BR, bradycardic response; a, P < 0.05 vs. Con; b, P < 0.05 vs. Inf*.

### Autonomic function

**Figure 2:**
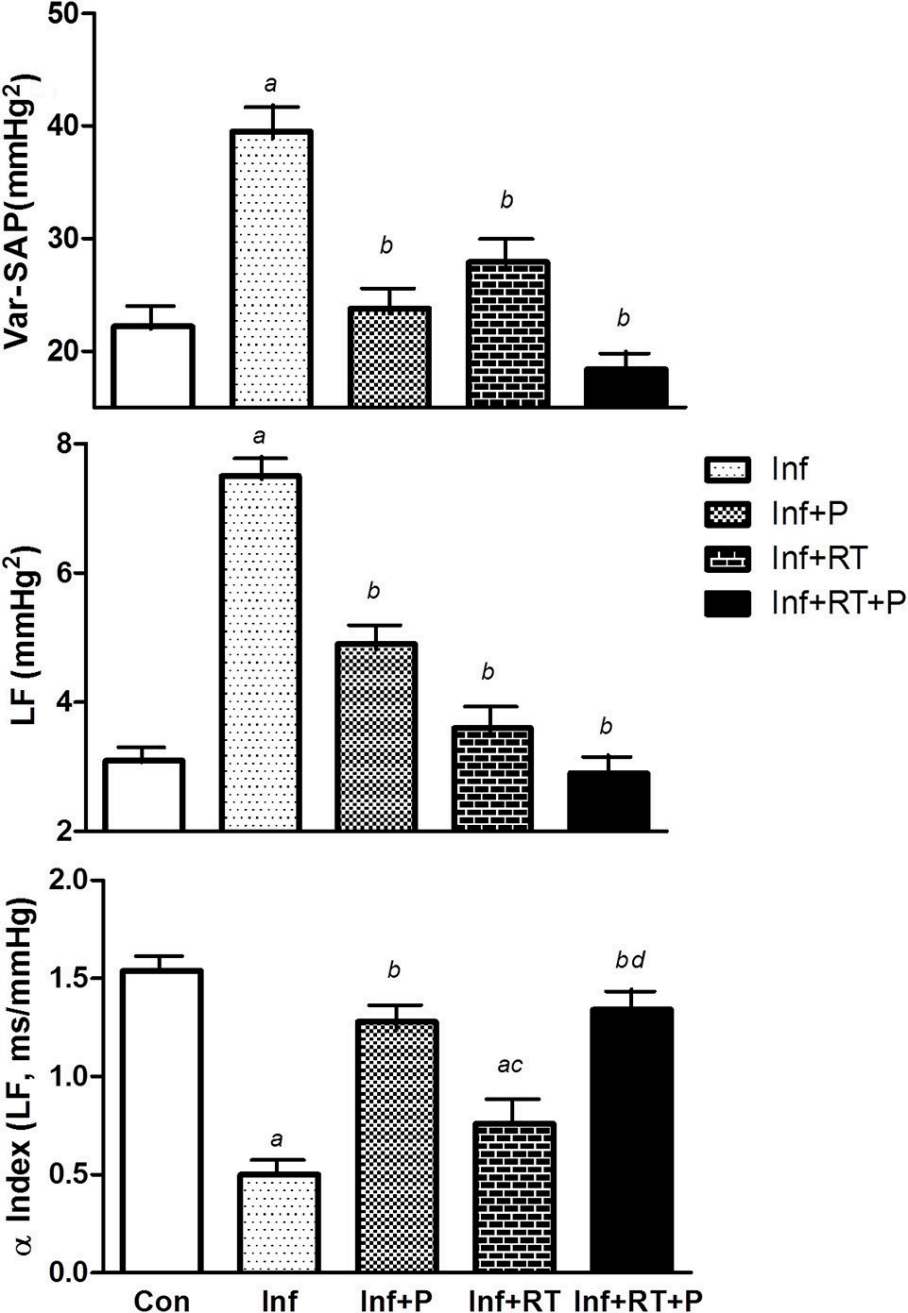
Systolic arterial pressure variability (Var-SAP). LF, Low frequency band. Control rats (Con); Sedentary infarcted rats (Inf); Sedentary infarcted rats treated with pyridostigmine (Inf+P); infarcted rats submitted to resistance exercise training (Inf+RT); and infarcted rats submitted to treatment with pyridostigmine and resistance exercise training (Inf+RT+P); Values expressed as mean ± SEM; *a, P* < 0.05 vs. Con; *b, P* < 0.05 vs. Inf; *c, P* < 0.05 vs. Inf+P; *d, P* < 0.05 vs. Inf+RT.

SAP variability parameters are presented in Figure 2. A significant impairment in Var-PAS, LF (mmHg2), and α Index (LF, ms/mmHg) were observed after MI. Resistance training and PYR treatment alone attenuated the deleterious effects of MI on Var-PI and LF (mmHg2), without further beneficial effects on these variables when PYR treatment and resistance training were combined (Inf+RT+P). A significant improvement in α Index was observed in Inf+P and Inf+RT+P. Nevertheless, a higher α Index was observed in Inf+RT+P when compared to Inf+RT.

Sympathetic tonus (ST), vagal tonus (VT), and intrinsic heart rate (IHR) are shown in Table 3. MI (Inf) elicited a marked increase in ST, whereas this effect was completely abolished in Inf+P and Inf+RT+P. IHR was not altered in the experimental groups, while PYR groups (Inf+P and Inf+RT+P) displayed increased VT followed by decreased ST when compared to Inf. However, VT values were higher after Inf+RT+P when compared to Inf+RT. On the other hand, VT was lower in Inf+RT when compared to Inf+P.

**Table 3:** Sympathetic (ST), vagal tonus (VT), and intrinsic heart rate (IHR).

	**Con**	**Inf**	**Inf+P**	**Inf+RT**	**Inf+RT+P**
ST (bpm)	40 ± 7	69 ± 8**a**	38 ± 6**b**	50 ± 4	32 ± 7**b**
VT (bpm)	64 ± 9	31 ± 7	87 ± 9**b**	49 ± 6**c**	89 ± 10**bd**
IHR (bpm)	355 ± 10	357 ± 18	375 ± 14	364 ± 9	372 ± 10

*Values expressed as mean ± SEM. Two-way ANOVA with Bonferroni posttest. a, P < 0.05 vs. Con; b, P < 0.05 vs. Inf; c, P < 0.05 vs. Inf+P; d, P < 0.05 vs. Inf+RT*.

### Systemic and tissue inflammatory profile

Table 4 shows the systemic inflammatory profile. The data obtained demonstrate that IL-6 levels were increased in the plasma of Inf group. On the other hand, treated and exercised groups showed a significant decrease in IL-6 levels. Regarding the anti-inflammatory profile, the PYR treatment was equally effective to elicit an increase in IL-10 levels in Inf+P and Inf+RT+P groups.

**Table 4:** Inflammatory markers.

	**Con**	**Inf**	**Inf+P**	**Inf+RT**	**Inf+RT+P**
**PLASMA**					
IL-6 (pg/mg prot)	1.67 ± 0.02	1.92 ± 0.06*a*	0.65 ± 0.03*ab*	1.57 ± 0.08*bc*	0.59 ± 0.03*abd*
IL-10 (pg/mg prot)	0.18 ± 0.02	0.12 ± 0.01	0.27 ± 0.05*b*	0.19 ± 0.04	0.29 ± 0.03*b*

*Values expressed as mean ± SEM. Two-way ANOVA with Bonferroni posttest. IL, Interleukin; a, P < 0.05 vs. Con; b, P < 0.05 vs. Inf; c, P < 0.05 vs. Inf+P; d, P < 0.05 vs. Inf+RT*.

**Figure 3:**
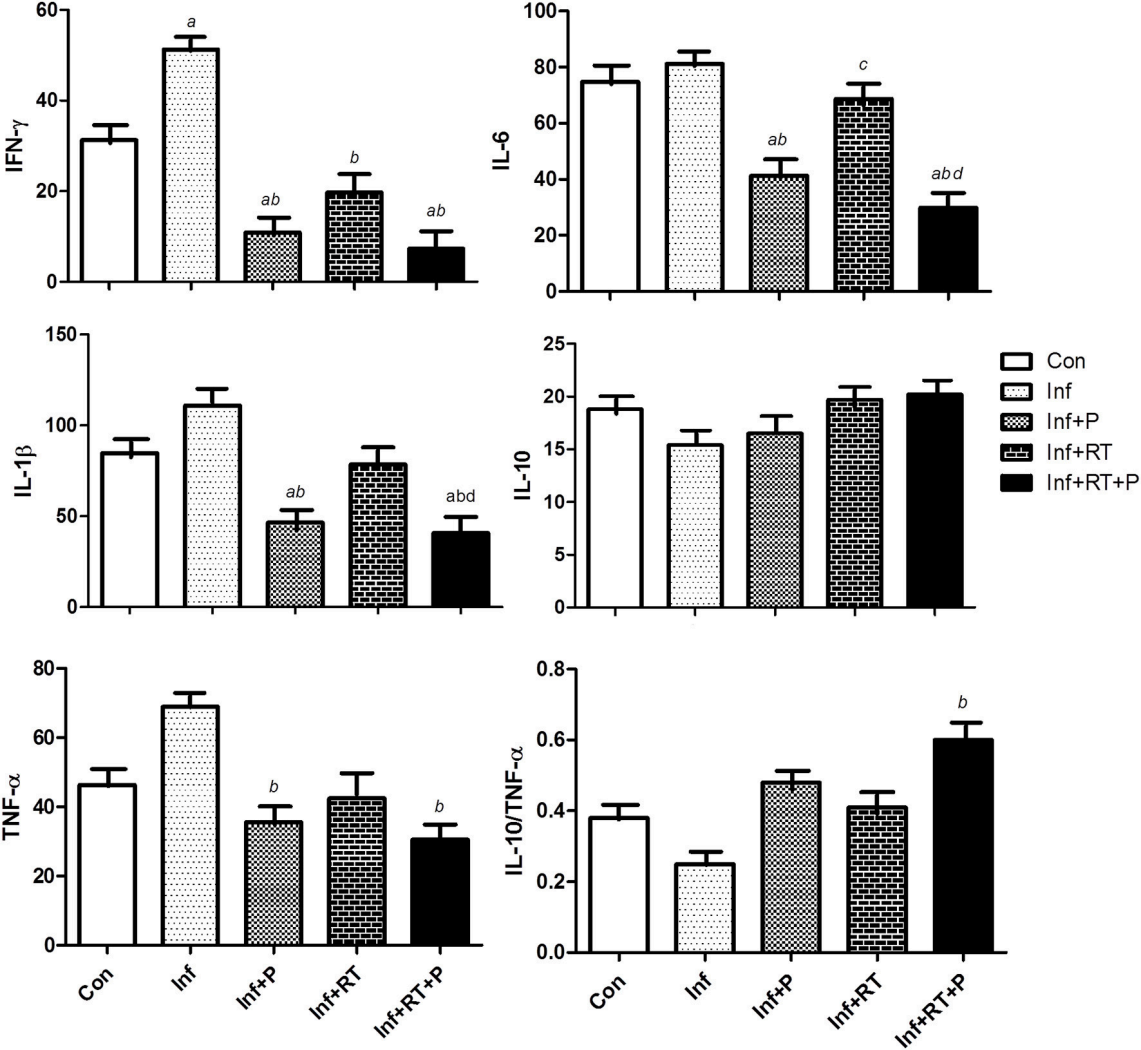
Left ventricle inflammatory profile expressed in pg/mg prot. Control rats (Con); Sedentary infarcted rats (Inf); Sedentary infarcted rats treated with pyridostigmine (Inf+P); infarcted rats submitted to resistance exercise training (Inf+RT); and infarcted rats submitted to treatment with pyridostigmine and resistance exercise training (Inf+RT+P); Values expressed as mean ± SEM; *a, P* < 0.05 vs. Con; *b, P* < 0.05 vs. Inf; *c, P* < 0.05 vs. Inf+P; *d, P* < 0.05 vs. Inf+RT.

Figure 3 shows the LV inflammatory profile. Inf group presented a significant increase in IFN-γ, which was completely abolished in Inf+P and Inf+RT, with no further beneficial results when the treatments were combined (Inf+RT+P). PYR treatment, either combined (Inf+RT+P) or not (Inf+P) with resistance training, elicited a decrease in the levels of IL-6, IL-1β, and TNF-α.

**Figure 4:**
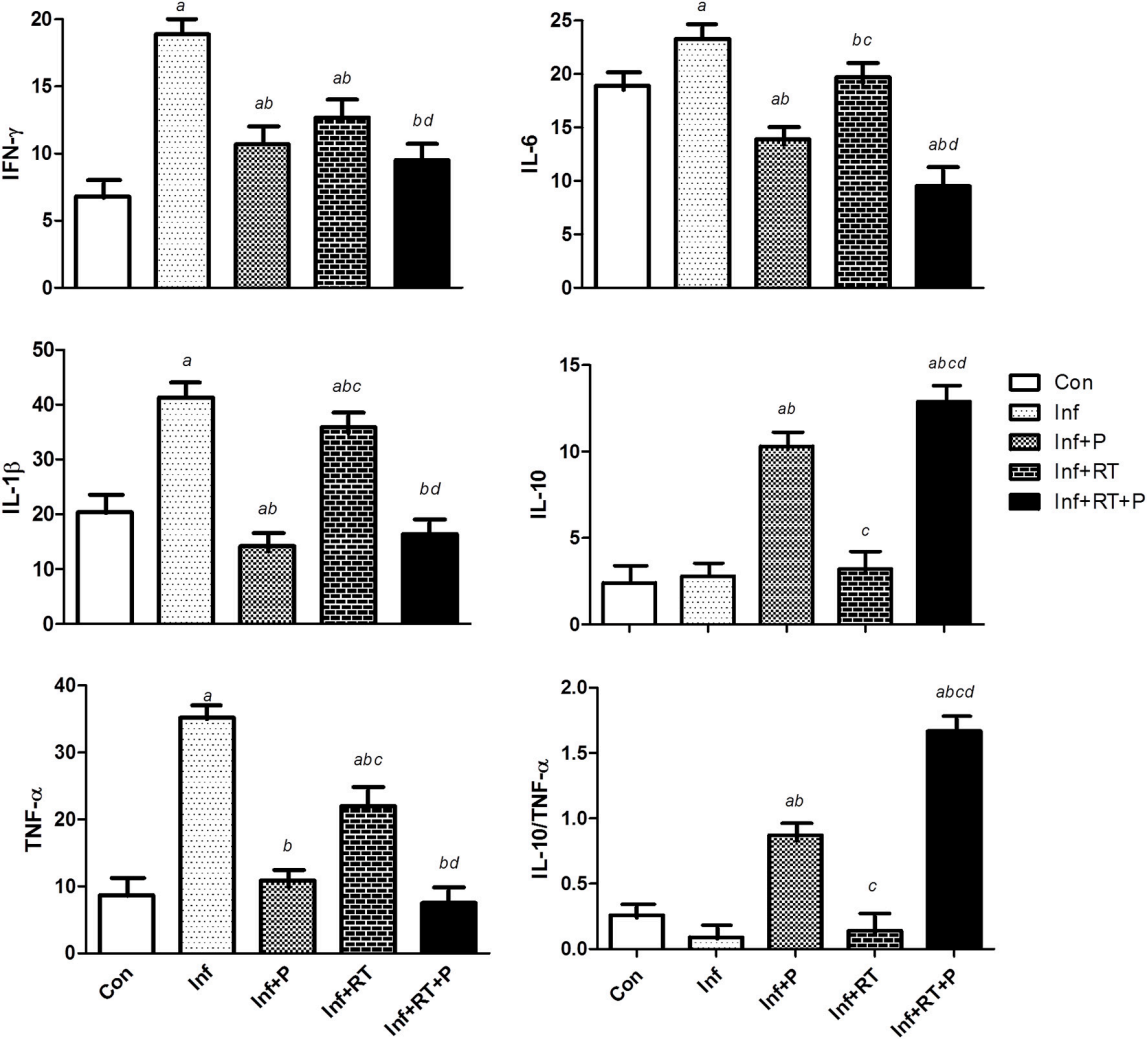
Muscle inflammatory profile expressed in pg/mg prot. Control rats (Con); Sedentary infarcted rats (Inf); Sedentary infarcted rats treated with pyridostigmine (Inf+P); infarcted rats submitted to resistance exercise training (Inf+RT); and infarcted rats submitted to treatment with pyridostigmine and resistance exercise training (Inf+RT+P); Values expressed as mean ± SEM; *a, P* < 0.05 vs. Con; *b, P* < 0.05 vs. Inf; *c, P* < 0.05 vs. Inf+P; *d, P* < 0.05 vs. Inf+RT.

Figure 4 shows the soleus muscle inflammatory profile. Inf group presented a significant increase in all inflammatory markers (i.e., IFN-γ, IL-6, IL-1β, and TNF-α). Inf+P, Inf+RT, and Inf+RT+P groups displayed important beneficial changes in this inflammatory parameters. However, only PYR treated groups (Inf+P and Inf+RT+P) demonstrated an increase in IL-10 and IL-10/TNF-α ratio levels. Furthermore, Inf+RT+P group presented lower levels of IFN-γ, IL-6, IL-1β, TNF-α when compared to Inf+RT and Inf, while the levels of IL-10 and IL-10/TNF-α ratio were higher when compared to all experimental groups.

## Discussion

The main findings of the present study indicate that RT and PYR treatment alone and in combination were able to improve the deleterious effects of MI. It is worth mentioning that the combination of RT and PYR treatment provided discreet additional effects on muscle strength, cardiac dimensions (i.e., MI akinetic area and LV mass), and autonomic function (i.e., α Index and VT) when compared to RT alone. In addition, Inf+RT+P group presented a higher expression of anti-inflammatory markers on skeletal muscle when compared to Inf+RT and Inf+P groups.

The findings of the present study are in line with some other studies observed in the literature. For instance, Grans et al. (2014) demonstrated that sedentary infarcted rats (Inf) presented a decrease of maximum load test (MLT) (~28%) when compared to non-infarcted rats. This finding is quite relevant, since MLT may reflect muscle strength and functional capacity, which has been described as an important predictor of mortality in MI patients when associated with other markers (Bucholz et al., 2016). Additionally, impaired muscle strength is one of the clinical symptoms of skeletal myopathy, a phenotypic condition characterized by several muscular abnormalities, such as muscle atrophy, exercise intolerance and motor disability, indicating a poor prognosis (Morley et al., 2006; von Haehling et al., 2013; Bacurau et al., 2016).

On the other hand, MLT was significantly increased in Inf+TR and Inf+TR+P. Other studies have corroborated these findings and have shown improved MLT in animal models of CVDs (i.e., hypertension and MI) after RT programs (Grans et al., 2014; Neves et al., 2016). Regarding PYR treatment, as expected, acetylcholinesterase activity was reduced in Inf+P when compared to non-treated groups, which paves the way to a large ACh concentration in the neuromuscular junction, possibly contributing to a better environment to improve MLT (Verma-Ahuja et al., 2000). However, the finding of our study does not lend support to that hypothesis, because the PYR treatment alone (Inf+P) did not elicit significant changes in MLT.

The effects of PYR treatment on muscle strength remain an understudied area and most research has failed to demonstrate improved physical capacity PYR-treated patients with and without muscle disorders submitted to PYR treatment (Breyer-Pfaff et al., 1990; Glikson et al., 1991). However, some studies have found that lower doses of PYR (1.2 mg/kg) may lead to an increase in the neuromuscular capacity of healthy mice (Verma-Ahuja et al., 2000). Therefore, as Inf+RT+P showed higher MLT values when compared to the other groups, we may hypothesize that PYR effects on muscle strength are dependent on a physical component.

As mentioned above, data of the present study indicate that 3 months of PYR treatment and RT were effective in promoting positive changes on echocardiographic parameters (LV dimension [LV mass and RWT] and function [EA ratio]), autonomic modulation and inflammatory profile of infarcted rats. Recent data from our group corroborate the results of the present study, e.g., the infarcted rats of Feriani et al. (2017) showed a significant improvement in cardiac and inflammatory parameters after 3 months of aerobic exercise and PYR treatment.

Furthermore, several other studies have demonstrated cardiovascular and immunological benefits of aerobic exercise for post-MI rats (Rondon et al., 2006; Flores et al., 2010; Jorge et al., 2011; Rodrigues et al., 2017). However, to the best of our knowledge, only one experiment so far had an exercise protocol based on RT (Grans et al., 2014). In their study, Grans et al. (2014) have observed changes in cardiovascular morphometry (i.e., LV mass, RWT) and diastolic function (E/A ratio).

In the present study, we demonstrated additional benefits of RT in the autonomic modulation and inflammatory profile when compared to the findings presented by Grans et al. (2014). The differences between the findings of the two studies are possibly due to the larger exercise volume (i.e., 15–20 repetitions versus 15 repetitions), which indicates that slight alterations in the prescription of resistance exercise may promote greater beneficial effects.

Regarding Inf+P, a number of studies are in line with our findings, since improved hemodynamic profile (La Fuente et al., 2013; Lataro et al., 2013; Durand et al., 2014; Corrêa et al., 2015; Rocha et al., 2016), autonomic function (La Fuente et al., 2013; Lataro et al., 2013; Durand et al., 2014; Rocha et al., 2016), BrS (La Fuente et al., 2013) and inflammatory state (Rocha et al., 2016; Feriani et al., 2017) have been observed after acute, short-term and long-term PYR treatments in infarcted rodents.

However, we should stress that results are still controversial regarding the effects of PYR on MI size (i.e., MI akinetic area) and on cardiac morphometry and function. While some studies seem to support the findings of the present study and indicate improvements in these variables (La Fuente et al., 2013; Lataro et al., 2013), others refuted them (Durand et al., 2014; Corrêa et al., 2015). The superior effects observed in the present study and in other investigations may result from the differences among the doses of PYR since we used a higher proportional dose of PYR when compared to Durand et al. (2014) and Corrêa et al. (2015).

Interestingly, autonomic dysfunction plays an essential role in the progressive deterioration of cardiac function and dimension after MI (Graham et al., 2002; La Fuente et al., 2013; Toschi-Dias et al., 2017). In fact, the hyperadrenergic state, which includes alterations in the afferent and efferent branches of the autonomic nervous system, is a crucial component in the development of heart failure from the progression of MI (La Rovere et al., 2002; Toschi-Dias et al., 2017). On the other hand, an improved parasympathetic activity seems to cooperate to improvements in these variables. In the experiment carried out by Lara et al. (2010), for example, mice with reduced expression of the vesicular ACh transporter (VAChT) showed marked LV dysfunction, followed by morphological disruption, and decreased calcium handling. However, 2 weeks of PYR treatment reversed this phenotype (Lara et al., 2010).

Therefore, the high doses of PYR used in the present study may simulate an increased parasympathetic activity to the heart, probably causing a higher concentration of ACh in the vicinity of the nerve endings in the heart. This would allow a greater coronary dilation through a nitric oxide-dependent mechanism (Parent et al., 1992; Quyyumi et al., 1995), thus preventing an increase in the MI area and avoiding major changes in cardiac structure and function. Moreover, Lataro et al. (2013) have demonstrated that PYR treatment may increase VEGF activity, and elicits a decrease in the diameter of cardiac myocytes and in the density of collagen, without changing MI area. This would indicate that an angiogenic process may play a role in the cardiac remodeling observed after PYR treatment.

In the present experiment, the combination of non-pharmacological (i.e., resistance exercise) and pharmacological treatment (i.e., PYR) elicited superior effects in muscle strength (i.e., MLT) and in skeletal muscle anti-inflammatory profile. These findings corroborate previous studies undertaken by our group (Feriani et al., 2017), and suggest that the beneficial effects of physical exercise and PYR treatment are not only observed after aerobic exercise.

Taken together, these findings indicate that the Inf+RT+P may elicit gains in the parasympathetic nervous system, from reflex mechanisms (BrS) to preganglionic cholinergic transmission in the two branches of the autonomic nervous system, which culminates in a low inflammatory profile and MI area (i.e., MI akinetic area). It should be emphasized that the inflammatory process is critical for cardiac remodeling after MI (Sutton and Sharpe, 2000). Furthermore, based on the *cholinergic anti-inflammatory pathway*, we might infer that the high concentration of ACh due to the treatments mediated the correlation between the parasympathetic nervous system and proinflammatory cytokines, thus preventing the deleterious effects of MI-induced cardiac remodeling in all experimental groups (Borovikova et al., 2000; Huston et al., 2006; Rosas-Ballina and Tracey, 2009; Rodrigues et al., 2014; Feriani et al., 2017).

Some limitations of the present study should be mentioned and addressed in future studies: lack of an extended evaluation of muscle strength, such as grip strength, and a histologic evaluation of the arrangement and aggregation of collagen fibers to confirm the MI area from echocardiographic results.

In conclusion, chronic PYR treatment associated or not with resistance training promoted important effects on functional, ventricular, autonomic and inflammatory parameters after myocardial infarction in rats. Importantly, the association between the therapies was able to improve the anti-inflammatory cytokine activation rather than attenuate pro-inflammatory cytokine on the studied tissues, which may occur in the light of cholinergic anti-inflammatory pathway.

## Author contributions

DF: contributed to conception and design of the work, acquisition of data, analysis, and interpretation of data, statistical analysis and draft the manuscript; HC-J: contributed to interpretation of data, statistical analysis and draft the manuscript; JdO: contributed to acquisition of data, analysis, and interpretation of data; MD: contributed to data analysis and interpretation; CM: contributed to analysis and interpretation of data; PD: contributed to acquisition of data, analysis, and interpretation of data; ÉC: contributed to acquisition of data, analysis, and interpretation of data; MI: contributed to conception and design of the work, analysis, and interpretation of data, statistical analysis and draft the manuscript; BR: contributed to conception and design of the work, interpretation of data, and draft the manuscript.

### Conflict of interest statement

The authors declare that the research was conducted in the absence of any commercial or financial relationships that could be construed as a potential conflict of interest.
