# Supplementary figures and images for: Pyridostigmine Improves the Effects of Resistance Exercise Training after Myocardial Infarction in Rats
Source: Front Physiol. 2018 Feb 12;9:53. doi: 10.3389/fphys.2018.00053 (PMC5816065; doi:10.3389/fphys.2018.00053)

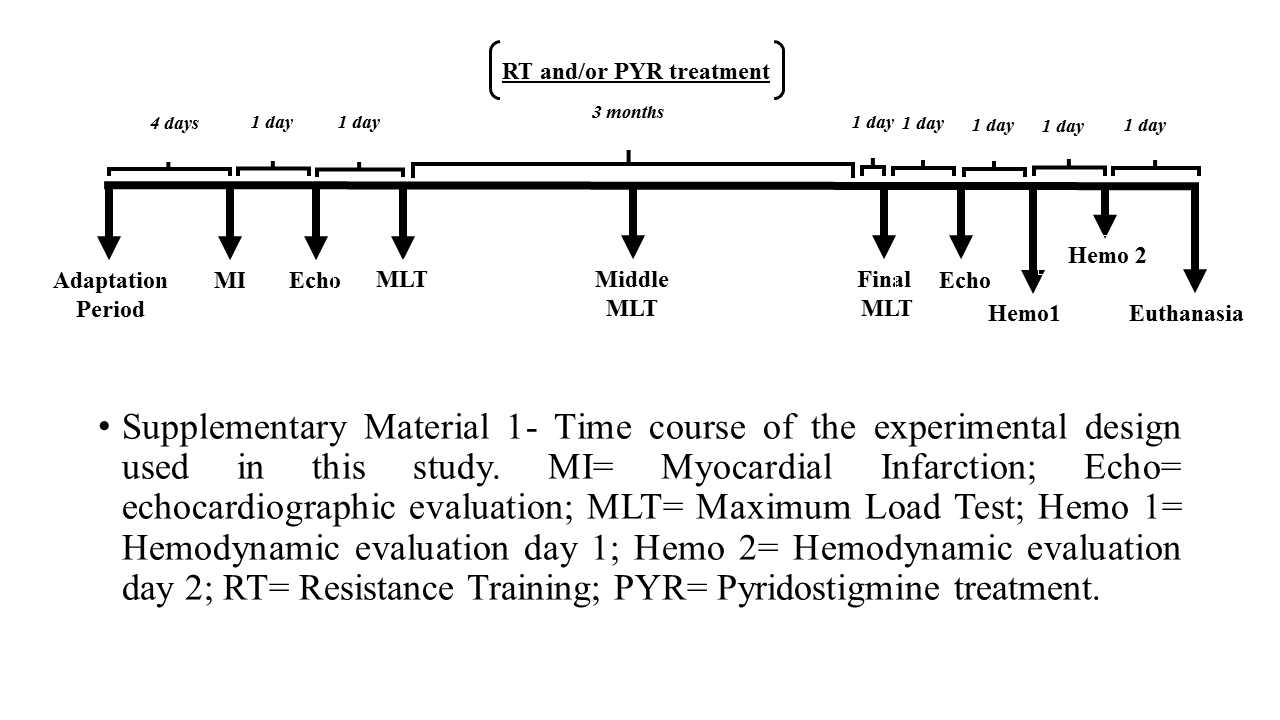

Supplement: Supplementary file 1 [file Image1.TIF]
